# Hypothyroidism-Related Cardiac Tamponade

**DOI:** 10.7759/cureus.18611

**Published:** 2021-10-08

**Authors:** Harsimran Kaur, Hyginus Chakwop Ngassa, Khaled A Elmenawi, Vishwanath Anil, Harpreet Gosal, Lubna Mohammed

**Affiliations:** 1 Family Medicine, California Institute of Behavioral Neurosciences & Psychology, Fairfield, USA; 2 Digestive Tract System, California Institute of Behavioral Neurosciences & Psychology, Fairfield, USA; 3 Surgery, California Institute of Behavioral Neurosciences & Psychology, Fairfield, USA; 4 Internal Medicine, California Institute of Behavioral Neurosciences & Psychology, Fairfield, USA; 5 Emergency Medicine, California Institute of Behavioral Neurosciences & Psychology, Fairfield, USA

**Keywords:** hypothyroidism, cardiac temponade, pericardial tamponde, hypothyroid pericardial effusion, chronic pericardial effusion

## Abstract

Thyroid dysfunction is a common incidental finding among healthy individuals. It can affect various organs of the body, including the heart. Among many other heart complications, it can lead to pericardial effusion by causing increased permeability of albumin across the pericardial membrane that leads to exudative pericardial effusion. In hypothyroidism, the fluid collection process occurs over a period of months, giving enough time for the pericardial membrane to stretch and accommodate the fluid within itself without causing any symptoms. Eventually, the pericardial membrane stretches to its maximum capacity and has no room to accommodate any more fluid, resulting in cardiac tamponade in the patients. Patients with hypothyroidism-related cardiac tamponade usually remain asymptomatic or present with atypical symptoms such as bradycardia and a normal heart rate or high blood pressure, and the diagnosis comes into light only when patients present to the hospital with hemodynamic instability. In these cases, echocardiography successfully detects large pericardial effusion with collapsed cardiac chambers. To treat hypothyroidism-related cardiac tamponade, treating the underlying condition has been very successful in the majority of the asymptomatic patients, but pericardiocentesis is required in emergencies to relieve symptoms of patients presenting with hemodynamic instability.

We believe hypothyroidism-related cardiac tamponade is a preventable condition if detected and treated in outpatient settings by family physicians. This will prevent occurrence of various complications arising from hypothyroidism, including pericardial effusion. This will lead to a better quality of life among patients with the added benefit of reduced health care burden due to reduced frequency of hospital admissions of acutely ill patients.

## Introduction and background

**Figure 1:**
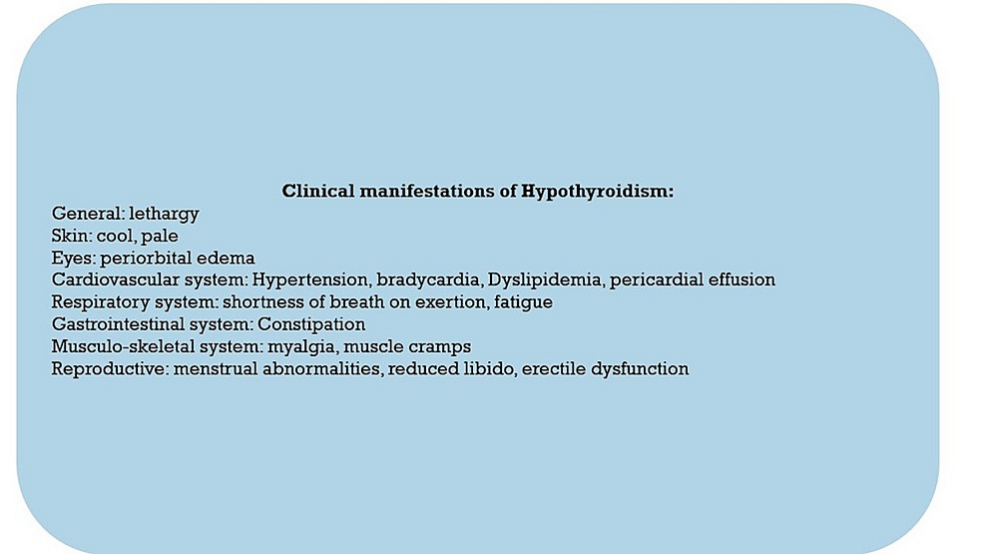
Clinical manifestations of Hypothyroidism

It is suggested that about 4% to 10% of the general population is affected by hypothyroidism [1]. Low thyroid hormone can affect almost every organ system of the body, leading to various clinical presentations (Figure 1). In the cardiovascular system (CVS), thyroid hormone plays a vital role in its development and function, mainly mediated by Triiodothyronine (T3). T3 causes increased tissue oxygen consumption (tissue thermogenesis), increased force of systolic contraction, and diastolic relaxation, along with an overall reduction in vascular resistance [2]. A lack of T3 in hypothyroidism leads to cardiovascular manifestations such as diastolic hypertension, sinus bradycardia, pericarditis, dyslipidemia, and pericardial effusion [3]. On physical examination, cardiovascular symptoms such as dyspnea, muffled heart sounds, non-pitting or pitting edema, and decreased exercise tolerance may be noted [4].

The structure that lines the heart and the proximal vessels is known as the pericardium. It is made up of two relatively avascular layer, the parietal and visceral pericardium, separated by a space that contains up to 50 ml of fluid, known as a pericardial fluid [5]. Pericardial effusion is a condition developed when there is increased accumulation of fluid in the pericardial cavity that can occur from various underlying pathologies such as a tumor, uremia, trauma, cardiac surgery, and hypothyroidism. In hypothyroidism, the incidence of pericardial effusion is about 3% to 6% in mild cases as compared to about 30% to 80 % in severe cases [6]. Typically pericardium is impermeable to proteins, whereas, in hypothyroidism, there is a rise in pericardial permeability and reduced lymphatic drainage of the albumin that causes leakage of proteins into the pericardial space, leading to accumulation of fluid in the pericardial space. Thus, the idea of exudative pericardial effusion is supported [5]. As the fluid accumulation is very slow, this gives the heart enough time to stretch and adapt to the change causing significant fluid accumulation without causing any hemodynamic compromise [7]. Although a larger volume of fluids can be accommodated in the pericardial space due to slow collection, at some volume, the pressure-volume curve will have a steep rise in pressure leading to tamponade physiology. Typical acute cardiac tamponade is a clinical diagnosis with clinical symptoms of hypotension, jugular vein distention, and tachycardia, known as Beck's triad. Contrary to this, hypothyroid patients present with bradycardia and a normal heart rate or high blood pressure. Pericardiocentesis is only required in hemodynamically unstable patients. However, hypothyroidism-related pericardial effusion may have echocardiographic evidence of tamponade [8,9].

This article will review the pathophysiology of cardiac tamponade in patients suffering from hypothyroidism and how it is different from cardiac tamponade occurring from other etiologies. We will also discuss the importance of early diagnosis and treatment of hypothyroidism to prevent life-threatening complications that may arise from severe hypothyroidism.

## Review

Pathophysiology of hypothyroidism associated pericardial effusion 

**Figure 2:**
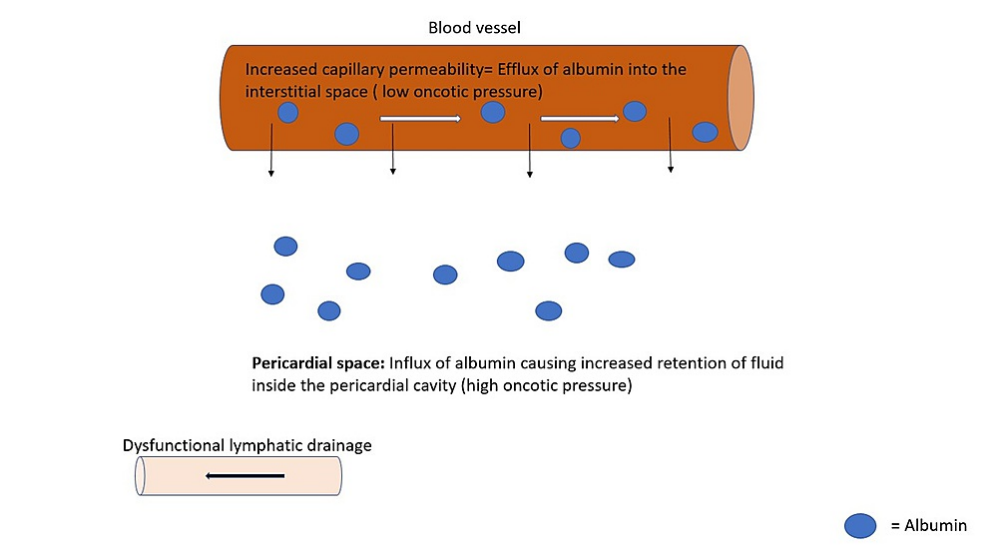
Pathophysiology of Hypothyroidism-related pericardial effusion

Physiologically, pericardial fluid is formed by ultrafiltration that occurs at the site of pericardial capillaries. Normally, hydrostatic pressure is higher in the arterioles than the pressure in the venules, and the colloid osmotic pressure created by the plasma proteins is essentially the same at both ends. Thus, most of the fluid gets reabsorbed at the venous end, and some of the retained fluid will be drained out via lymphatic drainage [5]. Typically, the pericardial space contains about 10 ml to 50 ml of pericardial fluid that acts as a lubricant between the pericardial layers. Any underlying condition can end up causing inflammation inside the pericardial cavity leading to increased fluid accumulation inside the cavity, thus causing pericardial effusion [10]. In hypothyroidism, there is low plasma volume, high vascular permeability, lower synthesis and catabolism rates of albumin, and prolonged passage time through the extravascular spaces causing increased albumin mass in the extravascular space [7]. This high vascular permeability to albumin increases the interstitial colloid pressure. As the colloid osmotic pressure gradient between the interstitial and the intravascular space reduces, there is a reduced fluid return to the capillaries at the venous end. Also, there is poor albumin drainage into the lymphatics that worsens the process, causing fluid retention inside the pericardial cavity [5] (Figure 2). 

Clinical presentation of hypothyroidism associated pericardial effusion

Hypothyroidism can cause various cardiac manifestations, such as bradycardia, atrial fibrillation, diastolic hypertension, varying degrees of atrioventricular (AV) block, prolonged QT interval leading to torsades de pointes, accelerated coronary artery disease, and pericardial effusion [7]. Clinically, hypothyroidism associated pericardial effusion can vary from being asymptomatic to presenting as cardiac tamponade, in which patients present with hemodynamic compromise [11]. Pericardial effusion can be classified based on onset (acute, subacute, and chronic), composition (transudative or exudative), and by its size, i.e., mild (<10 mm), moderate (10-20 mm), or large (>20 mm) [10]. The severity of pericardial effusion is dependent on the severity of the disease. Thus pericardial effusion is frequently found in myxedema, an advanced stage of hypothyroidism, and is rarely related to mild disease [12]. Also, the severity of the pericardial effusion depends on the speed of fluid accumulation in the pericardial sac. If it is getting collected over a short period, such as after trauma, the clinical presentation would be dramatic, i.e., even small amounts of blood over a short period can cause high pressure inside the pericardial cavity-causing hemodynamic instability in the patients. A slow accumulation of fluid, as in hypothyroidism, allows enough time for the pericardial membrane to stretch and accommodate the fluid. Thus, it takes weeks to months before symptoms start to appear, making cardiac tamponade a rare presentation in hypothyroidism [10]. Literature suggests that about half of all patients with chronic large pericardial effusion are asymptomatic [11]. When present, the patients may present with symptoms such as dyspnea (61.1%), cough (25%), and chest pain (13.9%) in the setting of clinical symptoms of severe hypothyroidism, including lethargy, facial swelling, dry skin with non-pitting edema, delayed relaxation of deep tendon reflexes. To evaluate the patients, a chest X-ray and ECG (electrocardiogram) are considered. Chest X-ray shows the enlarged cardiac silhouette, and electrocardiogram (ECG) shows changes, such as low voltage, sinus bradycardia, flattened T or inverted T waves, prolonged QTc, exposing the patient to high risk of developing torsades de pointes, ventricular tachycardia, and rarely, AV block. Abnormalities in the chest X-ray and the ECG are further investigated by an echocardiogram [8,10]. As per the guidelines, pericardial effusion should be managed by treating the underlying etiology as much as possible [10].

Clinical presentation of hypothyroidism associated cardiac tamponade

**Figure 3:**
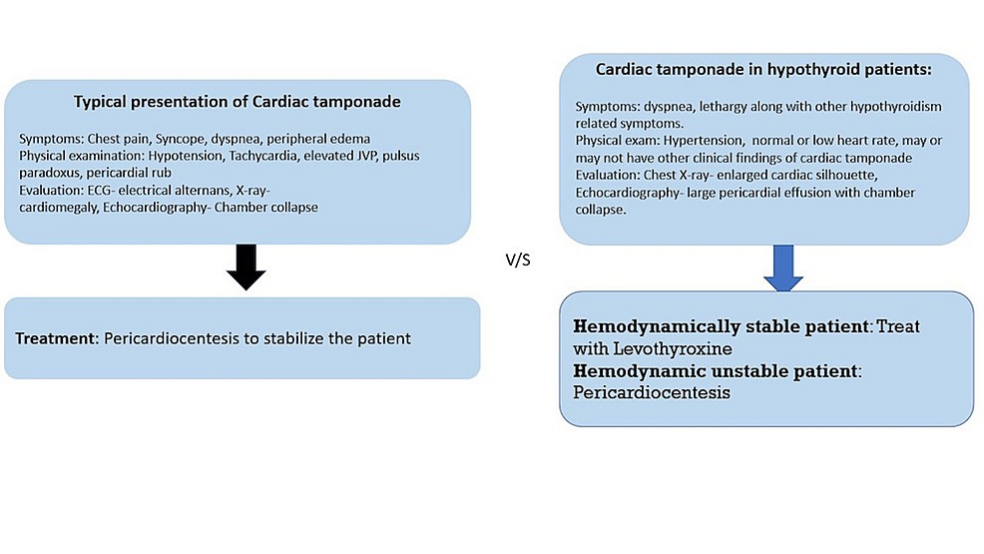
Clinical features of Hypothyroidism-related cardiac tamponade versus typical cardiac tamponade

Pericardial tamponade, also known as cardiac tamponade, is a condition in which there is an abnormally large amount of fluid accumulation in the pericardial cavity-causing increased intrapericardial pressure, which is then transmitted to cardiac chambers, thus reducing cardiac filling. Although pericardium has some degree of elasticity to accommodate some fluid, as in pericardial effusion, without showing clinical symptoms, once the limit is attained, it starts to compress all the cardiac chambers. Since the right atrium is thin-walled, it is not only the most vulnerable to compression by the pericardial fluid, but its increased pressure also affects the veno-atrial gradient that determines the cardiac filling [13]. This causes a hemodynamic compromise in the body, creating a shock-like state in the body. Pericardial tamponade is a clinical diagnosis that can be recognized by three signs, also known as Beck's triad. They include hypotension, jugular venous distention, and muffled heart sounds, along with pulses paradoxus. On investigation, ECG shows low-voltage QRS and electrical alternans due to the damping effect of pericardial fluid and swinging of heart inside the fluid. Echocardiograph is considered the single most useful diagnostic tool to identify the size, location, and degree of hemodynamic compromise. It is a medical emergency that needs quick removal of fluid from the pericardial cavity. This is done with pericardiocentesis [14] (Figure 3).

On the contrary, cardiac tamponade may present differently in patients with a history of hypothyroidism. To analyze the management plan of hypothyroidism-related cardiac tamponade, we collected case reports of eight different patients. Patients in Table 1 were treated medically with Levothyroxine. On the other hand, patients in Table 2 required pericardiocenteses. Baldwin et al. [8] compared three unique cases of cardiac tamponade in patients with a history of hypothyroidism. All of them were successfully treated without invasive interventions with Levothyroxine. Chou et al. [15] reported a patient with a history of laryngectomy. He was suspected of having hypothyroidism due to his history of neck surgery. Once the diagnosis was confirmed, he was successfully treated medically with oral thyroxine for his massive pericardial effusion. Although hypothyroidism-related cardiac tamponade can be managed medically by treating underlying hypothyroidism, pericardiocentesis may be required in some cases. Wang et al. in 2010 studied 36 patients in total in which only eight patients (22.2 %) had both clinical and echocardiographic findings of tamponade and were treated by pericardiocentesis [16]. We described four cases of hypothyroidism-related cardiac tamponade that required pericardiocentesis in order to stabilize the patient. Once stabilized, patients were managed medically with Levothyroxine to prevent recurrence of pericardial effusion (Table 2). Once hemodynamic stability is obtained, the patient should be further investigated to look for the underlying cause to prevent reoccurrence. Also, the pericardial fluid analysis showed yellow fluid without any bacteria or malignant cells present in it [9,12,17,18]. Alexander first described this in 1919, as the pericardial effusion of "gold-paint" appearance due to the presence of cholesterol in the fluid with no bacteria in it [19].

**Table 1:** Hypothyroidism-related cardiac tamponade being with Levothyroxine YO: year old, BP: blood pressure, HR: heart rate, SpO2: oxygen saturation, T: temperature, PE: physical examination, LE: lower extremities, UE: upper extremities, CVS: cardiovascular system, TFT: thyroid function tests, EKG: electrocardiogram, CXR: chest X-ray, TTE: transthoracic echocardiogram, RV: right ventricle, RA: right atrium, DTR: deep tendon reflexes, JVP: jugular venous pressure. TEE: transesophageal echocardiogram. TSH: thyroid stimulating hormone. 2L O2: two liters of oxygen.

Research Study​	Past Medical History​	Clinical Presentation​	Physical Findings​	Investigations​	Management Plan​
1. Baldwin et al. [8]​ ​	hypertension​	63 YO female presented after mechanical fall, reported to have functional decline over the past three months​	Vital signs:​ BP: 149/85, HR: 56, SpO2: 97% on room air, T: 95.8°F​ PE: right-sided facial droop, and bilateral pitting edema (LE), CVS: no significant findings.​	Labs: TFT: hypothyroidism, rhadomyolysis.​ EKG: sinus bradycardia with T-wave flattening, TTE: large pericardial effusion with RV collapse ​	Intravenous Levothyroxine​
2. Baldwin et al. [8]​ ​	rheumatoid arthritis, polymyositis, interstitial lung disease, and hypothyroidism​	61 YO female presented with three days of generalized weakness​	Vitals: BP: 150/113, HR: 83, SpO2: 100% on room air, T: 98.0°F​ PE: slurred speech, weakness (UE,LE), non-pitting edema, dry skin, and delayed DTR. CVS: elevated JVP, muffled heart sounds​ ​	Labs: TFT: hypothyroidism, EKG: low voltage with T-wave flattening, TTE: large pericardial effusion along with RA/RV diastolic collapse suggestive of tamponade, pericardiocentesis: straw coloured fluid​	Intravenous Levothyroxine​ ​
3. Baldwin et al. [8]​ ​	hypothyroidism​	66 YO female presented with severe lethargy, cough, and mild dyspnea​	Vitals: BP: 162/90, HR: 89, SpO2: 97% 2L O2, T: 97.3°F​ PE: facial swelling, dry skin, delayed DTR. CVS: distant heart sounds​	Labs: TFT: hypothyroidism. ​CXR: enlargement of cardiac silhouette, EKG: T-wave flattening. TEE: large pericardial effusion along with RA/RV diastolic collapse ​	IV Levothyroxine. Pericardiocentesis only to decompress left main stem bronchus​
4. Chou et al. [15]​	laryngeal carcinoma​ surgical treated with total laryngectomy and adjuvant radiotherapy ​	68 YO male presented with bradycardia, postural hypotension, mild exertional dyspnea, and occasional chest tightness for three days.​	Vitals: BP: 92/57, HR: 50, T: 34.5°C​ PE: dry skin​ CVS: distant heart sounds without murmurs.​	Labs: High TSH, low T3 and T4 suggestive of severe hypothyroidism​ CXR: enlarged cardiac silhouette, EKG: sinus bradycardia, TTE: moderate to large pericardial effusion​	Oral Thyroxine​

**Table 2:** Hypothyroidism-related cardiac tamponade requiring pericardiocentesis YO: year old, SOB: shortness of breath, BP: blood pressure, HR: heart rate, SpO2: oxygen saturation, T: temperature, PE: physical examination, LE: lower extremities, UE: upper extremities, CVS: cardiovascular system, BNP: B-type natriuretic peptide, TFT: thyroid function tests, EKG: electrocardiogram, CXR: chest X-ray, TTE: transthoracic echocardiogram, RV: right ventricle, RA: right atrium, DTR: deep tendon reflexes, JVP: jugular venous pressure. TEE: transesophageal echocardiogram. TSH: thyroid stimulating hormone.

Research Study​	Past Medical History ​	Clinical Presentation​	Physical Examination Findings​	Investigation Findings​	Management Plan​
1. Butala et al. [9]​	Diabetes mellitus, Hypertension​	42 YO female presented with generalized weakness, abdominal pain and episodic SOB​	Vitals: BP: 202/117, HR: 74, afebrile but became hemodynamically unstable shortly after the admission.​ PE: lethargy, bilateral leg oedema over the ankles and pretibial areas.​ CVS: distant heart sounds​	Labs: normal BNP, CXR: cardiomegaly, TTE: large pericardial effusion with diastolic collapse of RV.​ Pericardiocentesis: negative for bacteria and malignancy cells.​ ​	Pericardiocentesis that did not resolve effusion​. Patient was later found to be hypothyroid, she was treated with levothyroxine for recurrent pericardial effusion.​
2. Patil et al. [12]​	Previously healthy​	45 YO female with two months history of malaise, lethargy, slow speech, edema of the face and extremities and progressive weight gain for the past two months, presented with SOB for two days.​	Vitals: BP: 90/52, HR: 74 , T: 36°C, SpO2: 84% on room air​ PE: facial edema, coarse hair, dry skin, an engorged jugular vein, mild pallor, and non-pitting edema of the extremities. CVS: distant heart sounds, pulsus paradoxus present.​	CXR: cardiomegaly, EKG: low-voltage pattern with electrical alternans, TTE: massive pericardial effusion with early RV diastolic collapse.​ Pericardiocentesis: yellow-golden​	Pericardiocentesis​: patient was later found to be hypothyroid, she was treated with thyroxine.​ Follow-up after three months showed complete resolution of effusion.​
3. Retnam et al. [17]​	Pellagra​	40 YO male presented with progressive distension of the abdomen, puffiness of face, edema of feet and exertional dyspnea​	Vitals: BP: 90/70, HR: 60, T: 37°C,​ PE: puffy face, loss of lateral third of the eyebrow, dry and course skin with areas of lichenification. GI: signs of free fluid in abdomen, CVS: raised JVP, distant heart sounds​	Labs: hypothyroidism,​ CXR: cardiomegaly,​ EKG: sinus bradycardia, low QRS voltage, TTE: cardiac tamponade.​ Pericardiocentesis: yellow and opalescent contained fibrin with no organism ​	Pericardiocentesis to relieve acute symptoms. ​Patient was then started on thyroxine to treat the underlying cause of tamponade.​ Follow-up after few weeks showed complete resolution.​
4. Usalan et al. [18]​	Not known​	65 YO woman with a one-month history of weakness and malaise was admitted to hospital because of chest pain, confusion, sweating and severe dyspnea​	Vitals: BP: 80/60, HR: 62, T: 35.7°C​, PE: hepatomegaly​ CVS: jugular venous distension, pulsus paradoxus, Kussmaul's sign​	CXR: cardiomegaly, EKG: reduction in amplitude of the QRS complex and sinus rhythm, TTE: massive pericardial effusion with RA/RV diastolic collapse.​ Pericardiocentesis: negative for bacteria and malignant cells.​	Pericardiocentesis:​ patient was later found to be hypothyroid, she was treated with levothyroxine. Follow-up after two months showed minimal pericardial effusion on TTE.​

Hypothyroidism presenting with hypertensive emergency and intracranial hemorrhage

Hypothyroidism may also present as a hypertensive crisis, which is an infrequent complication but can happen. Chui et al. [2] reported that a 20-year-old male patient presented with a one-month history of intermittent blurred vision, which was getting worse. His vitals were as follows: BP 224/140 mm Hg, HR 62 beats per minute. On cardiovascular (CVS) examination, JVP was not elevated, and no pulsus paradoxus or cardiac murmurs were present. Neurological examination showed bitemporal hemianopsia, and fundoscopic examination of the eyes showed papilledema and hypertensive retinopathy. On investigation, neuroimaging and ECG findings were insignificant. An echocardiogram showed large circumferential pericardial effusion. Laboratory work showed hypothyroidism, and he was started on high-dose thyroid replacement and anti-hypertensive agents for his hypertension. However, he became short of breath; pericardiocentesis was performed to relieve his symptoms. After he became stable, he was discharged on oral thyroid replacement therapy. On follow-up appointment, his high blood pressure had resolved with normalization of echocardiogram findings. Hwang et al. [20] reported a 46-year-old female who presented to the hospital with left arm weakness and dysarthria that had started 30 minutes ago. On general examination, the patient had a puffy face, and there was generalized edema. Cardiovascular examination was insignificant. Her vitals were as follows, BP 213/124mm Hg, HR 60 beats per minute. Neurological examination showed dysarthria and weakness in the left arm of motor grade 1-2. On investigation, intracranial CT showed hemorrhage. Chest X-ray showed a "water bottle" sign, indicating cardiomyopathy and transthoracic echocardiogram showed a large amount of pericardial fluid. She was being managed for her high blood pressure to prevent worsening neurological symptoms, but she started to have shortness of breath and reduced awareness. Urgent pericardiocentesis was performed to relieve her symptoms. On further investigation, her TSH was elevated. She was started on Synthroid along with anti-hypertensive agents. On follow-up after six months, her dose of anti-hypertensive medication was reduced due to normalization of thyroid hormone level. It was concluded that severe hypothyroidism might present with hypertensive crisis and hemorrhage, and these life-threatening situations can be well prevented by managing the patient's underlying medical condition.

Limitations

In this review article, we studied eight patients thoroughly from the literature to draw our conclusion on how we can prevent life-threatening cardiac temponade in hypothyroidism patients. To check accuracy of this study, we encourage future researchers to conduct larger observational studies and come up with recommendations for regular follow-up of hypothyroidism patients and strategies to create awareness about the nature of medical condition among patients.

## Conclusions

As hypothyroidism can cause symptoms of almost all body systems, patients may present with vague symptoms, due to which diagnosis may be missed in many patients. Additionally, there are no recommendations for screening among the general population for thyroid dysfunction, which together contribute to delayed diagnosis among patients. It is evident from the above-mentioned examples that patients who missed the diagnosis of hypothyroidism ended up needing pericardiocentesis in the hospitals. It should also be noted that thyroid dysfunction can also present as worsening of a patient's chronic condition such as diabetes, chronic heart failure, depression, and fatigue, etc. Thus, thyroid function should be routinely tested as part of their routine blood work. Once diagnosed, patients should be regularly followed up by their family physician during the time they are undergoing treatment and thereafter to see if they continue to remain euthyroid. The frequency of follow-up may vary depending on various factors such as the severity of disease, presence of multiple comorbidities and compliance to the medication, in which case the patients should be seen more frequently and should be assessed if they are candidate for referral to a specialist. Emphasis should also be made to educate the patients about the nature of their disease and the complication that may arise if they are left untreated. This way, a family physician can play a huge role in significantly reducing hypothyroidism-related cardiovascular complications and can help prevent hospital admissions among these patients.
